# Field Response of the Twig Beetle, *Pityophthorus pubescens,* to the Aggregation Pheromone, (E)-(+)-pityol, is not Inhibited by (E)-(-)-pityol, and Evidence of Monogyny

**DOI:** 10.1673/031.013.1501

**Published:** 2013-02-21

**Authors:** Sergio López, Juan Carlos Iturrondobeitia, Arturo Goldarazena

**Affiliations:** 1 NEIKER-TECNALIA, Basque Institute of Agricultural Research and Development, Department of Plant Production and Protection. Arkaute, 46. E-01080 Vitoria Spain.; 2 Department of Zoology and Animal Cell Biology, University of Basque Country, Sarriena s/n. E-48940, Leioa, Spain.

**Keywords:** flight response, galleries, reproduction, Scolytinae

## Abstract

Field studies were conducted to determine the flight response of the twig beetle *Pityophthorus pubescens* (Marsham) (Coleoptera: Curculionidae: Scolytinae) to the potential aggregation pheromone component, (*E*)-(+)-pityol, and its racemic form, (*E*)-(±)-pityol, in different *Pinus* spp. L. (Pinales: Pinaceae) stands in the Basque Country (Northern Spain). Both (*E*)-(+)-pityol and (*E*)-(±)-pityol equally attracted more males than females, and they were all significantly different from the blank controls. Data about *P. pubescens* gallery systems in naturally infested *P. radiata* D. Don branches are also provided. The presence of one male and one female was the most common gallery habitation found in debarked twigs. Current results suggest that *P. pubescens* may be monogynous and females might mediate the aggregation of mating partners.

## Introduction

Pitch canker disease, caused by the fungal agent *Fusarium circinatum* (= *F. subglutinans* f.sp. *pini*) (Niremberg and O'Donnell) (Hypocreales: Nectriaceae), has spread throughout the world. It is well reported that several insect species, mainly bark beetles, are capable of transmitting the pathogen (Fox et al. 1991; Hoover et al. 1995). Twig beetles of genus *Pityophthorus* Eichhoff (Coleoptera: Curculionidae: Scolytinae) have been traditionally considered as a secondary bark beetles group, due to their life habits and development in branches of dead and decaying trees (Bright 1981; Wood 1982). However, *P. setosus* and *P. carmeli* in California (Storer et al. 2004; Sakamoto et al. 2007) and *P. pubescens* (Marsham) in the Basque Country (northern Spain) (Romón et al. 2007) have been associated with *F. circinatum.*

Little is known about the biology of *P. pubescens.* According to previous research, it is considered as the only species of the genus inhabiting *P. radiata* stands of the Basque Country (López et al. 2007; López 2010), and it breeds in a broad number of *Pinus* L. (Pinales: Pinaceae) species (Balachowsky 1949; Pfeffer, 1976). It seems to show preference for host fresh tissues, according to the avoidance it exhibits to 4,6,6-trimethylbicyclo-[3.1.1]hept-3-en-2-one ((1*S*, 4*S*)-(-)-- verbenone) (Romón et al. 2007). However, there is a lack of knowledge about the biology and chemical ecology of the species.

Evidence of (2*R,* 5*S*)-2-(1-hydroxy-1-methylethyl)-5-methyltetrahydrofuran [hereafter (*E*)-(+)-pityol] as an aggregation pheromone component has been presented by López et al. (2011). Results obtained via effluvia collection revealed that both sexes emit (*E*)-pityol, although neither enantiomeric composition nor differences in emissions between sexes were elucidated. Moreover, behavioral assays in Y-tube olfactometer showed a stronger attractive effect on males (López et al. 2011). Taking into account these previous results, the main objective of our study was to determine the response of *P. pubescens* to (*E*)-(+)-pityol and the racemic mixture in different *Pinus* spp. stands of the Basque Country, in order to provide insight into the aggregation behavior of the species, and in the second instance, to infer the breeding behavior of the species through the observation of gallery systems.

## Materials and Methods

### Chemicals

Both (*E*)-(+)*-*pityol (99% chemical purity) and the racemic mixture (99%) were kindly synthesized by Prof. W. Francke (Institute of Organic Chemistry, University of Hamburg, Hamburg, Germany), following the methodology described in Mori and Puapoomchareon (1987).

### Field assays

**Figure 1.**
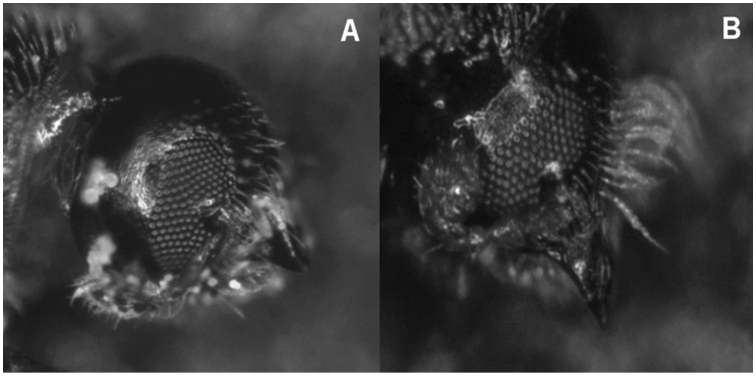
*Pityophthorus pubescens* head. (A) Male. (B) Female. Note the pubescent frons. High quality figures are available online.

Two assays were carried out in four different *Pinus* spp. mature stands (ca. 30–35 years) of similar area in the Basque Country during 2011. The first trapping assay was conducted from 1 May to 30 June 2011, whereas the second one took place from 15 July to 15 September. This temporal frame was set due to the flight periods of *P. pubescens* in these months (S. López unpublished data). Three eight-unit Lindgren multiple funnel traps (Econex S.L., www.e-econex.com), 106 cm height, were placed in each stand. Sampling localities and their geographic coordinates are detailed in Table 1. Each trap was hung between two adjacent trees, with the top of the trap at 2 m above the ground and the distance between traps at least 75 m. One trap was set as a blank control, whereas the other two traps were baited either with (*E*)-(+)-pityol or the racemic mixture. Releasing devices consisted of one 1.5-mL capped plastic microcentrifuge vial (VRW International, www.vwr.com) containing 2.5 µl of the attractant. After sealing them, caps were pierced with a number 2, heated insect pin (Dallara et al. 2000). The vials were refilled every week, and traps were re-randomized every week. Fifty mL of propylene glycol (propane 1,2-diol) (99%) (Panreac, www.panreac.com) were added to each trap cup in order to kill and preserve captured insects. Insects caught were removed every week during the two months (n = 8 weeks, considered as replicates), taken to the laboratory, and identified under a Leica MZ95 stereo microscope (Leica Microsystems, www.leica-microsystems.com) with the aid of identification keys (Balachowsky 1949; Pfeifer 1976). Gender was determined based on the presence of yellowish hairs on the frons, a typical characteristic of females (Pfeffer 1976) (Figure 1). Voucher specimens were deposited at the Entomology Collection of the NEIKER-Basque Institute for Agricultural Research and Development, Basque Country, Spain.

**Table 1.**
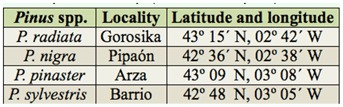
*Pinus* spp. stands sampled during field trapping with (*E*)-(+)-pityol and racemic (*E*)-pityol in different localities in the Basque Country (Northern Spain).

### Branch dissection

Naturally-pruned branches (ca. 10 cm length and 5 mm diameter) were collected from a 30–40 year-old *P. radiata* stand, located at Gorosika (43° 15′ N, 02° 42′ W), Basque Country. Every week, from the beginning of May until the end of June, 40 naturally pruned branches were randomly picked up from the ground. Branches were dissected with a scalpel under a stereo microscope. Photographs were taken with a DFC300 (Leica Microsystems) camera attached to the stereo microscope.

### Statistical analysis

Field-trapping mean catches of males and females were compared independently for each *Pinus* species and subjected to a two-way ANOVA analysis (with sex and pheromone taken as factors), followed by Tukey *post-hoc* tests at a significance level of α *=* 0.05. A square root transformation was used to normalize the data and correct the heteroscedasticity. All the analyses were performed with the statistical software SPSS 2004 SYSTAT statistical package (version 13.0, SPSS, www-01.ibm.com/software/analytics/spss)

## Results

### Field assays

Apart from *P. pubescens,* no other bark beetle species were found in multiple funnel traps. A total of 9,121 *P. pubescens* were caught during field trapping from May to June, whereas 8,241 were trapped from July to September. Mean sex ratio, expressed as the percentage of males caught, for each sampling period and *Pinus* spp. stand is shown in Table 2. Twoway ANOVA revealed significant differences within sexes in all *Pinus* spp. stands, both in May–June (*P. radiata F*5,42 = 326.12, *p* < 0.001; *P. sylvestris F*5,42= 89.27, *p* < 0.001; *P. pinaster* F5,42 = 221.37, *p* < 0.001; *P. nigra F*5,42= 51.43, *p* < 0.001) and July–September (*P. radiata F*5,42= 340.84, *p* < 0.001; *P. sylvestris F*5,42 = 76.30, *p* < 0.001; *P. pinaster F*5,42 = 235.75, *p* < 0.001; *P. nigra F*5,42= 45.99, *p* < 0.001). In all these cases male catches were significantly higher. Significant differences were found between (*E*)-pityol baited traps and the blank control, although there were no differences between (*E*)-{+)*-*pityol and the racemic mixture in both sexes and experiments (Table 3, 4).

**Table 2.**
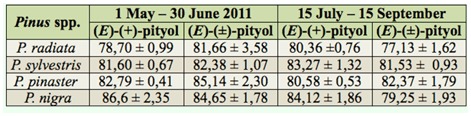
Mean sex ratio data (± SEM), expressed as percentage of male catches in (E)-pityol baited multiple-funnel traps, for both field-trapping periods and *Pinus* spp. stand.

### Branch dissection

**Figure 2.**
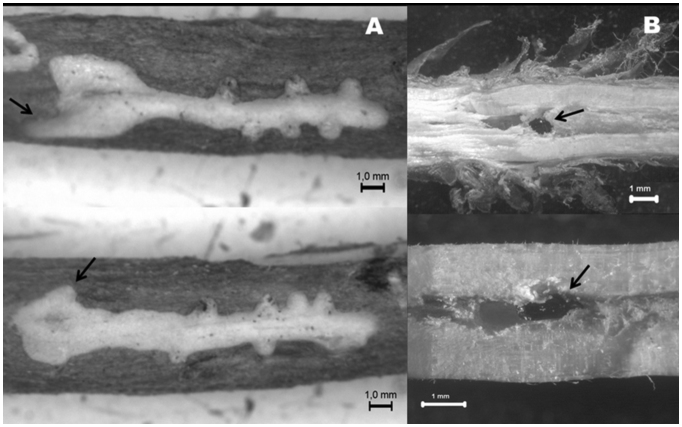
*Pityophthorus pubescens* gallery pattern. (A) Inner-bark excavated galleries, with egg niches across the gallery. Arrows indicate entrance holes. (B) Female *P. pubescens* boring into the pith (arrows). High quality figures are available online.

Of 147 examined galleries that contained at least one *P. pubescens,* 108 (73.46%) contained both a male and female, whereas 24 (16.32%) contained only one female. In addition, seven males (4.76%) were found alone in a gallery, four galleries (2.72%) were found to have two males and one female, three had two males and two females (2.04%), and only one had one male and two females (0.68%). It is remarkable that all beetles initiating galleries (24 females and seven males) were found until mid-May. On the contrary, galleries containing more than a single *P. pubescens* were collected at later dates. 102 galleries with a pair of beetles of both sexes had been excavated in the inner bark, with parental galleries showing a longitudinal pattern (Figure 2A). However, six galleries containing a pair were observed in the pith of relatively fresh branches (Figure 2B). Pair-occupied parental galleries showed a mean length of 6.57 ± 2.90 mm (range 2.28–14.19 mm).

**Table 3.**
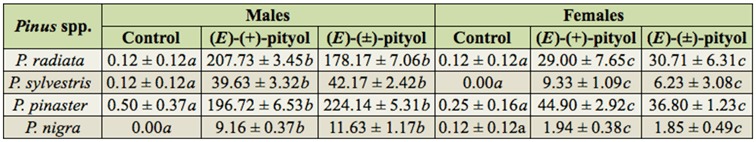
Mean captures (± SEM) per week (n = 8 weeks/replicates) of male and female *Pityophthorus pubescens* in Lindgren funnel traps baited with (*E*)-(+)-pityol and racemic (*E*)-pityol in different *Pinus* spp. stands of the Basque Country (Northern Spain) ( 1 May—30 June 2011). Means within the same row followed by a different letter are significantly different at *p* ≤ 0.05 (Two-way ANOVA, followed by Tukey *post-hoc* tests).

**Table 4.**
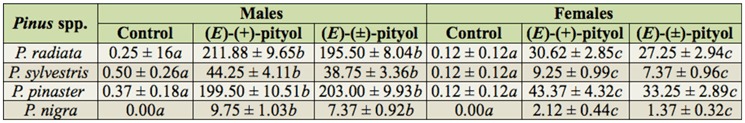
Mean captures (± SEM) per week (n = 8 weeks/replicates) of male and female *Pityophthorus pubescens* in Lindgren funnel traps baited with (*E*)-(+)-pityol and racemic (*E*)-pityol in different *Pinus* spp. stands of the Basque Country (Northern Spain) ( 15 July—15 September 2011). Means within the same row followed by a different letter are significantly different at *p* < 0.05 (Two-way ANOVA, followed by Tukey *post-hoc* tests).

## Discussion

Here, we provide evidence of the attractive effect of (*E*)-(+)-pityol and the racemic mixture on *P. pubescens* in field trapping. (*E*)-(+)-pityol is considered as a component of the aggregation pheromone of some species of the genus *Pityophthorus.* It was first isolated from male *P. pityographus* (Francke et al. 1987), and later from male *P. carmeli,* female *P. setosus*, and female *P. nitidulus* (Dallara et al. 2000). (*E*)-(+)-pityol is also known to be the female-produced sex pheromone of cone pine beetles, *Conophthorus resinosae* (Pierce et al. 1995), *C. coniperda* (Birgersson et al. 1995), and *C. ponderosae* (Miller et al. 2000). In all these cone beetle species, males are attracted strongly to (*E*)-(+)-pityol. In current work, attraction mediated by (*E*)-(+)-pityol and the racemic mixture did not differ, with both attracting significantly more male *P. pubescens* than females.

In contrast, the negative enantiomer of (*E*)pityol appears to be inactive to *Pityophthorus* spp. (Francke et al. 1987; Dallara et al. 2000; W. Francke, personal communication). European *P. pityographus* did not response to the mixture of racemic grandisol and (*E*)-(-)-pityol, whereas an attractive effect was observed when replacing (*E*)-(-)-pityol with (*E*)*-*(+)-pityol (Francke et al. 1987). Neither *P. setosus* nor *P. carmeli* were attracted to (*E*)-(-)-pityol (Dallara et al. 2000), and (*E*)-(-)-pityol did not interrupt the response of *P. setosus* to (*E*)-(+)-pityol (Dallara et al. 2000). Thus, this evidence and previous significant results with (*E*)-(+)-pityol and the racemic mixture (López et al. 2011) led to focus our work on these two compounds.

Traditionally, monogamy for these beetles has been defined as the reproductive behavior in which either sex may initiate the boring of a new gallery, and both sexes usually participate in the construction and care of the galleries (Wood 1982). The initiating sex is always constant. However, this term was redefined by Kirkendall (1983), who suggested a different point of view based on the mating system. According to Kirkendall, monogamy is considered as a mating system characterized by the presence of males in the gallery system until females finish ovipositing, whereas monogyny terminology is applied to those gallery systems made by the work of a single female (Kirkendall 1983). Hereafter, this terminology will be followed. Several genera of subtribe Pityophthorina, including *Pityophthorus* spp. complex, are characterized as harem polygynous, according to their mating system (Kirkendall 1983). Almost all Nearctic *Pityophthorus* species are considered within this category, but with some exceptions. Bright (1981) suggested that monogyny may be present in pith borer species, such as *P. opaculus* and *P. puberulus*, and evidence of monogyny has been found in *P. setosus* (Dallara et al. 2000), *P. orarius*, and *P. fivazi* (Kirkendall 1983). In addition, monogyny seems to be present in Palearctic *P. henscheli*, *P. morosovi, P. traegardhi,* and *P. carniolicus* (Pfeffer 1976). This reproductive behavior is also present in the closely related genus *Conophthorus* (Wood 1982). On the contrary, harem polygyny is characterized by (1) the presence of some female in a gallery system in which one female is already present and (2) a male-initiated gallery system and male-production of semiochemicals primarily attractive to females, whereas monogyny systems are based on a gallery system made by the work of a single female (Kirkendall 1983). Thus, taking into account these assertions, if one considered *P. pubescens* as polygynous, one would expect to find various females in a multiple arm gallery system around a nuptial chamber. Interestingly, our results are in contrast to this expected idea, and support the monogynous mating system hypothesis. First, parental galleries occupied by one female and one male consisted of a single and longitudinal gallery, with the absence of any star-shape nuptial chamber. This pattern of gallery habitation is similar to that found for *P. setosus* (Dallara 1997). In contrast, only one mother gallery was occupied by one male and two females, a typical pattern of polygynous. Secondly, most of the beetles that had initiated gallery construction were females, while gallery initiation was found for only seven males. It has been demonstrated that both sexes of *P. setosus* and *P. carmeli* are capable of tunneling in phloem of *P. radiata* bolts (Dallara et al. 2000). However, the proportion of male *P. carmeli* constructing galleries was found to be significantly higher than that of females. Conversely, a significantly greater proportion of female *P. setosus* excavated (Dallara et al. 2000). Our data are consistent with *P. setosus,* and could be evidence for considering females as the gallery initiation sex.

The sex ratio of beetles that responded to semiochemical trapping was strongly male-biased. Male catches either with the chiral (*E*)-pityol or the racemic mixture represented more than the 80% of the total catches in almost all *Pinus* spp. stands. These results are consistent with Dallara et al. (2000). In polygynous *P. carmeli,* females responded stronger than males to (*E*)-(+)-pityol and (5*S*, 7*S*)*-*(-)-7-methyl-1,6-dioxaspiro(4.5)decane (*E*)-(- )conophthorin) mixture, whereas in monogynous *P. setosus,* attraction to (*E*)-(+)-pityol alone was significantly higher in males than in females. Moreover, male *P. pityographus,* another polygynous species, is only attracted by the combination of *cis-*1-(2-hydroxyethyl)-1-methyl-2-(1-methyl-ethenyl)-cyclobutane ((±)-grandisol) and (*E*)-(+)-pityol (Francke et al. 1987). In fact, aggregation in these two polygynous species is not mediated in absence of any of the aggregation component. Our results resemble those observed for *P. setosus,* with a single component attracting significantly more opposite conspecifics.

According to our results, we propose the following host-attacking and mating sequence. Pioneering females would perform with the suitable-host seeking proccess and start the gallery construction. Then, the emission of (*E*)-pityol would attract males and a single male subsequently would enter the gallery initiated by each female. However, since both sexes appear to emit (*E*)-pityol, the biological implication of male-produced (*E*)-pityol remains still unclear. In *Conophthorus* spp., males emit (*E*)-(-)-conopthorin in order to repel and warn rival males (Birgersson et al. 1995). Nevertheless, it is unlikely that male *P. pubescens* would emit (*E*)-pityol as a warning signal for conspecific males, since we have demonstrated that (*E*)-pityol elicits a significant attractive effect on males. Thus, male-produced (*E*)-pityol requires further studies to disclose its biological activity.

In those gallery systems in which a pair of opposite conspecifics was present, eggs were observed. They had been deposited in small individual chambers on both sides of the mother gallery, and they were greyish, with oblong shape, 0.354 mm average length (range 0.303–0.399 mm, n = 58), and 0.545 mm average width (range 0.501–0.595, n = 58). Larvae were also present in some galleries, but no measures or biological data were taken. The mite species *Pyemotes parviscolyti* (Acari: Pyemotidae) was found in 56 breeding galleries. Female *P. parviscolyti* were found feeding on larvae and eggs of *P. pubescens.* Phoretomorphic females were also observed attached to the first pair of coxae of both sexes of *P. pubescens.*

In light of our results, we suggest using (*E*)-(+)-pityol or the more readily available and economical racemic mixture as a useful tool for monitoring populations of *P. pubescens* in *Pinus* spp. stands. In addition, it could even be used as a potential mate-finding disruptor in field due to its strong attractive effect on males. This kind of strategy was previously used with *C. coniperda,* with promising results (Trudel et al. 2004). There are commercially available devices containing the racemic mixture (95.4%) with a 0.2 mg/day release rate, which might correspond to about the equivalent of 200 female *C. coniperda* (De Groot and DeBarr 1998), and it could be as effective as the synthetic compounds. Further field studies should be carried out to determine if mate disruption in *P. pubescens* would be feasible.
